# *hsdS*, Belonging to the Type I Restriction-Modification System, Contributes to the *Streptococcus suis* Serotype 2 Survival Ability in Phagocytes

**DOI:** 10.3389/fmicb.2017.01524

**Published:** 2017-08-09

**Authors:** Bin Xu, Ping Zhang, Weiyi Li, Rui Liu, Jinsheng Tang, Hongjie Fan

**Affiliations:** ^1^College of Veterinary Medicine, Nanjing Agricultural University Nanjing, China; ^2^Poultry Institute, Chinese Academy of Agricultural Sciences Yangzhou, China; ^3^Jiangsu Co-innovation Center for Prevention and Control of Important Animal Infectious Diseases and Zoonoses Yangzhou, China

**Keywords:** *Streptococcus suis* serotype 2, host specificity determinant specificity subunit, peptidoglycan-binding protein, anti-phagocytosis, survival ability, reactive oxygen species, nitric oxide, TNF-α

## Abstract

*Streptococcus suis* serotype 2 (SS2) is an important zoonotic agent in swine and humans. Anti-phagocytosis and survival in phagocytic cells and whole blood is essential for bacteria to be pathogenic. In this study, the host specificity determinant specificity subunit (coded by *hsdS*) of the Type I Restriction-Modification system and two peptidoglycan-binding proteins (coded by *lysM* and *lysM*′, respectively), which were simultaneously found to be subjected to transcript-level influence by *hsdS*, were identified to facilitate the anti-phagocytosis of SS2 to a microglia cell line BV2. Furthermore, they significantly enhanced its survival in BV2, whole blood, and a peroxidation environment (H_2_O_2_) (*p* < 0.05), yet not in the acidic condition based on statistical analysis of the characteristic differences between gene mutants and wild-type SS2. In contrast, another specificity subunit, coded by *hsdS*′, that belonged to the same Type I Restriction-Modification system, only significantly reduced the survival ability of SS2 in the acidic condition when in the form of a gene-deleted mutant (*p* < 0.05), but it did not significantly influence the survival ability in other conditions mentioned above or have enhanced anti-phagocytosis action when compared with wild-type SS2. In addition, the mutation of *hsdS* significantly enhanced the secretion of nitric oxide and TNF-α by BV2 with SS2 incubation (*p* < 0.05). The SS2 was tested, and it failed to stimulate BV2 to produce IFN-γ. These results demonstrated that *hsdS* contributed to bacterial anti-phagocytosis and survival in adverse host environments through positively impacting the transcription of two peptidoglycan-binding protein genes, enhancing resistance to reactive oxygen species, and reducing the secretion of TNF-α and nitric oxide by phagocytes. These findings revealed new mechanisms of SS2 pathogenesis.

## Introduction

*Streptococcus suis* serotype 2 (SS2), one of the most pathogenic and frequently isolated serotypes among 33 serotypes of *Streptococcus suis* around the world, is responsible for various diseases in swine and humans, including meningitis, septicemia, etc. (Greeff et al., 2011). It has caused severe economic losses in the porcine industry and endangered public health security in several Asian and European countries as well as in North and South America, Australia and New Zealand (Wertheim et al., 2009; Gottschalk et al., 2010). Although numerous virulence factors have been reported, the pathogenic mechanism of SS2 has not yet been clearly established (Fittipaldi et al., 2017).

**Figure 1:**
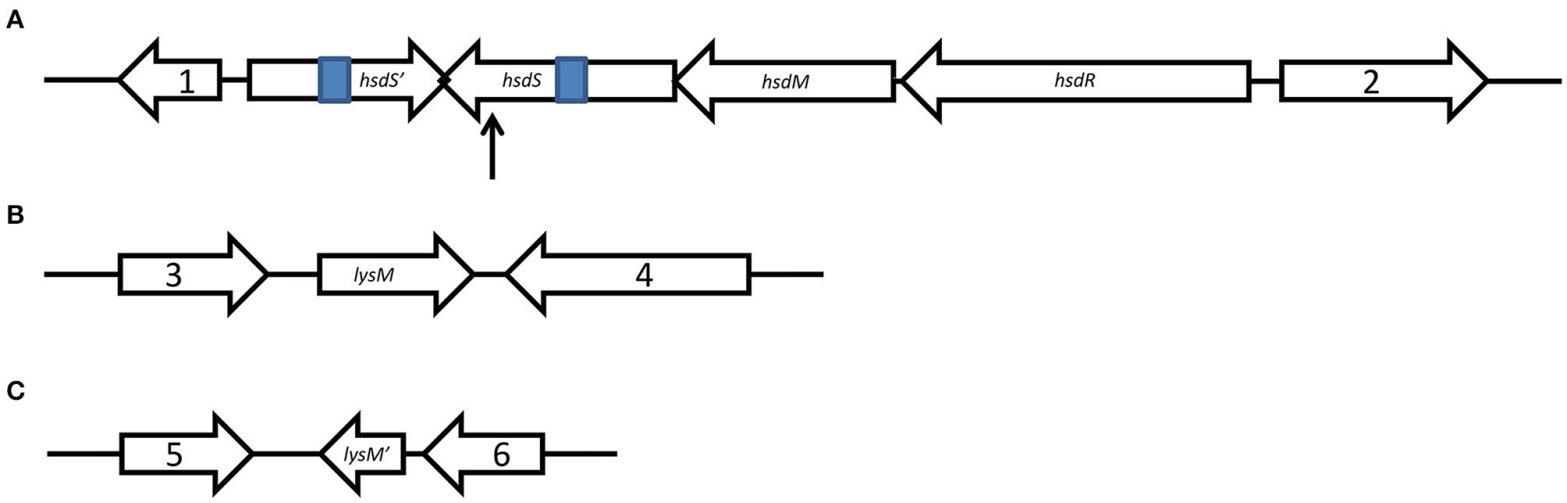
Schematic representation of genetic organization. Schematic maps of the SsuZY05719II locus **(A)**, as well as *lysM* **(B)**, and *lysM*′ **(C)** with their upstream and downstream genes were showed. Gene 1, encoding a ribose-5-phosphate isomerase, locus_tag ZY05719_RS06845; gene 2, encoding a tRNA uridine-5-carboxymethylaminomethyl synthesis GTPase MnmE, locus_tag ZY05719_RS06870; gene 3, encoding a oxidoreductase, locus_tag ZY05719_RS01205; gene 4, encoding a glucohydrolase, locus_tag ZY05719_RS01215; gene 5, encoding a L-serine dehydratase, locus_tag ZY05719_RS10230; and gene 6, encoding a transporter, locus_tag ZY05719_RS10240. The 307 bp of invert repeats are shown in blue. The arrow is pointing to the TnYLB-1 insertion site.

An insertion mutation of SS2 ZY05719, obtained by a TnYLB-1 insertion from pMar4s, demonstrated that the capacity of anti-phagocytosis (This term means phagocytosis resistance, which comes from ref Bergman et al., 2009; Yamaguchi et al., 2009; Hsieh et al., 2016) compared with the wild-type strain was significantly reduced in our earlier research (Liu et al., 2016). The TnYLB-1 insertion site was between the T^1384^ and A^1385^ bases in the open reading frame (ORF) of the host specificity determinant specificity subunit (*hsdS*) gene (locus_tag ZY05719_RS06855) (Figure 1A). The Type I Restriction–Modification (RM) system contains three host specificity determinant (*hsd*) genes coding a modification subunit (M), which protects the host DNA through DNA methylation by methyltransferase activity; a restriction subunit (R), which digests the foreign DNA by restriction endonuclease activity; and a specificity subunit (S), which determines the recognition sequence of both restriction and modification activities by the central repeat region and two target recognition domains (TRDs) (Price et al., 1989; Furuta et al., 2011; Loenen et al., 2014). In addition to protecting against invading DNA, the RM system can function as a mobile genetic element stabilizer, gene expression regulator, and virulence enhancing factor (Vasu and Nagaraja, 2013). The SS2 ZY05719 genome (GenBank accession code NZ_CP007497) contains two Type I RM systems. We propose to name one of the Type I RM systems as SsuZY05719II according to the REBASE criteria (http://rebase.neb.com). SsuZY05719II is found between a ribose-5-phosphate isomerase gene and a tRNA modification GTPase gene, including one *hsdM*, one *hsdR*, and two different intact *hsdS*, named *hsdS* and *hsdS*′, respectively (Figure 1A). SsuZY05719II lacks an integrase or a recombinase. The two *hsdS* each has two TRDs, a central repeat region, and 5′ end ribosomal binding sites and promoters, which were predicted through Softberry (http://www.softberry.com). PCR using extracted genomic DNA as a template shows that no recombination occurred in the exchange of TRDs between the two *hsdS* (data not shown). All three aspects indicated the random recombination through the exchange of TRDs between two *hsdS*, just as occurred in the Type I SpnD39III RM system of *Streptococcus pneumonia* D39 strain (Manso et al., 2014), is unlikely to exist. The findings indicated these two *hsdS* may each has a unique regulatory mechanism that needs to be elucidated. Additionally, the systematic study of the regulation of virulence in the Type I RM system in SS2 has not previously been performed (Willemse and Schultsz, 2016), and that type of systematic study is the goal of this research.

Meanwhile, we found two SS2 ZY05719 genes, which code peptidoglycan-binding proteins, were both positively impacted on transcriptional levels by *hsdS*. Each protein contains a LysM domain, which is a general peptidoglycan-binding module. Then these two genes were named *lysM* and *lysM*′, respectively. As one of the most common components in the cell surface proteins of bacteria, the LysM domain was originally identified in bacterial lysins that degrade cell walls (Bateman and Bycroft, 2000), and they were named for the lysin motif (Ponting et al., 1999). Later studies reported some proteins that harbored the LysM domain do not always have lyase activity, but they are involved in virulence, such as *Escherichia coli* Intimin (Jerse et al., 1990), *Staphylococcus aureus* IgG binding protein A (Uhlén et al., 1984), and Listeria monocytogenes p60 protein (Dussurget et al., 2004). Here, a systematic study of *hsdS, hsdS*′, *lysM* and *lysM*′ functions in SS2 ZY05719 on anti-phagocytosis and survival in adverse environments were performed for SS2.

## Materials and methods

### Bacterial strains, plasmids, cell lines, and growth conditions

The bacterial strains and plasmids used in this study are listed in Table 1.

**Table 1:** Summary of bacterial strains and plasmids used in this study.

**Bacterial strain or plasmid**	**Notable characteristic (s)^a^**	**Source of reference**
**BACTERIAL STRAINS**		
*E. coli* DH5α	Plasmid cloning host	Purchased from Invitrogen
ZY05719	Virulent strain of SS2 isolated from a dead pig	Lab collection
ZY05719 M*hsdS*	ZY05719 transposon mutants obtained by TnYLB-1 insertion within the open reading frame (ORF) of gene *hsdS*	Lab collection Liu et al., 2016
ZY05719 Δ*hsdS′*	Isogenic *hsdS′* mutant of strain ZY05719	This study
ZY05719 Δ*lysM*	Isogenic *lysM* mutant of strain ZY05719	This study
ZY05719 Δ*lysM′*	Isogenic *lysM′* mutant of strain ZY05719	This study
ZY05719 CM*hsdS*	Complemented strain of ZY05719 M*hsdS*, Spc^r^	This study
ZY05719 CΔ*hsdS*′	Complemented strain of ZY05719 Δ*hsdS*′, Spc^r^	This study
**PLASMIDS**		
pSET4s	*S. suis* thermosensitive suicide vector, Spc^r^	Takamatsu et al., 2001a
pSET2	*E. coli*- *Streptococcus* shuttle cloning vector, Spc^r^	Takamatsu et al., 2001b
pSET4s::*hsdS′* UD^b^	Recombinant vector designed to knock out gene *hsdS′*, Spc^r^	This study
pSET4s::*lysM* UD^b^	Recombinant vector designed to knock out gene *lysM*, Spc^r^	This study
pSET4s::*lysM′* UD^b^	Recombinant vector designed to knock out gene *lysM′*, Spc^r^	This study
pSET2::*hsdS*	pSET2 containing *hsdS* in the EcoR I-BamH I restriction sites, Spc^r^	This study
pSET2::*hsdS′*	pSET2 containing *hsdS′* in the EcoR I-BamH I restriction sites, Spc^r^	This study

a *Spc^r^, spectinomycin*.

b *UD, upstream and downstream flanking sequences of a gene*.

ZY05719, a virulent strain of SS2, was isolated and identified from a dead pig during an outbreak in Sichuan, China in 2005. SS2 ZY05719 and its derivative mutants were cultured at 37°C in Todd-Hewitt (TH, BD, Cat.No.249240) broth or on TH agar. *Escherichia coli* strain DH5α was used as a host for plasmids and cultured in Luria-Bertani (LB, Sigma-Aldrich, Cat.No.L3147) broth or on LB agar at 37°C. Once the bacteria contained plasmids, 50 or 100 μg mL^−1^ spectinomycin was added to the culture medium for *E. coli* or SS2, respectively. The murine microglia cell line BV2 was purchased from the Cell Resource Center, IBMS, CAMS/PUMC. As a valid substitute for primary microglia cells, a special type of phagocyte, this cell line exhibits morphological and functional characteristics of microglia (Blasi et al., 1990; Bocchini et al., 1992; Henn et al., 2009). It was cultured in Dulbecco's modified Eagle medium (DMEM) high glucose (GIBCO, Cat.No.11965) supplemented with 10% fetal bovine serum (GIBCO, Cat.No.10099-141) at 37°C under 5% CO_2_.

### Deletion and functional complementation of genes

The primers used in these experiments are listed in Table 2.

**Table 2:** Primers used in this study.

**Name**	**Oligonucleotide sequence (5′–3′)^a^**	**Product**
**CONSTRUCTION OF RECOMBINANT PLASMID**		
hsdS′-UF	GCCGGATCCCCAATAAAGAGACCGTGT	Upstream of *hsdS′*
hsdS′-UR	CTATCGAGAAATGAAAGATTCAATTTCTTGGACTGT	Upstream of *hsdS′*
hsdS′-DF	ACAGTCCAAGAAATTGAATCTTTCATTTCTCGATAG	Downstream of *hsdS′*
hsdS′-DR	GCCGTCGACGGTTTTGGAGATACACTT	Downstream of *hsdS′*
hsdS′-F	GGAGTGGGTGAGGTTGGGT	Part of *hsdS′*
hsdS′-R	TCAACTTTGTTTTTGGAAT	Part of *hsdS′*
lysM-UF	GCGGTCGACGAGGAAAATCAGGTCGTCTTG	Upstream of *hsdS*
lysM-UR	GCTGAAACGGTTATTTTTGTAAGTAATCTTATTCCTTCATTATTTTTG	Upstream of *hsdS*
lysM-DF	CAAAAATAATGAAGGAATAAGATTACTTACAAAAATAACCGTTTCAGC	Downstream of *hsdS*
lysM-DR	CGGGAATTCTCAATGCCTACCAGATGCTC	Downstream of *hsdS*
lysM-F	CTTCATTGGCTCTTTCCCTC	Part of *hsdS*
lysM-R	ACGGTCTGGCATCAGGTTC	Part of *hsdS*
lysM′-UF	GCGGTCGACAATGCCAAAATGGGCTTAGT	Upstream of *hsdM*
lysM′-UR	ATGACAGAAAGGAATGTGACTATTGATAAATTAGAAGAGGTCTAGCACG	Upstream of *hsdM*
lysM′-DF	CGTGCTAGACCTCTTCTAATTTATCAATAGTCACATTCCTTTCTGTCAT	Downstream of *hsdM*
lysM′-DR	CGGGAATTCGAAGAGGCTTTGTATTTGAACG	Downstream of *hsdM*
lysM′-F	GCATCTGCTACACGCTCTT	Part of *hsdM*
lysM′-R	CAACTATTGCTGGTTTGGT	Part of *hsdM*
hsdS-CF	GCCGGATCCGACGAAAAAAACAGAAGA	*hsdS* and its promoter
hsdS-CR	GCCGAATTCTCAGTAATAAAGTTGGGC	*hsdS* and its promoter
hsdS′-CF	GCCGGATCCGTCTCCTTTGAAGTAAAACGAA	*hsdS′* and its promoter
hsdS′-CR	GCCGAATTCTTCAGGATTATTGATACCTATTTCT	*hsdS′* and its promoter
**qPCR**		
ZY05719_RS01205-F	TGTCTACGCTCTGTCCTTTG	Part of ZY05719_RS01205
ZY05719_RS01205-R	AAATCCCTACCTGCTCGTC	Part of ZY05719_RS01205
lysM-F	CTTGCTGGCGAAAATAAATC	Part of *lysM*
lysM-R	CAGTTGCCTGAACGGTCTC	Part of *lysM*
ZY05719_RS01215-F	TACCGATCTCCTCACCCAT	Part of ZY05719_RS01215
ZY05719_RS01215-R	AACAACCACGACCAACCTC	Part of ZY05719_RS01215
ZY05719_RS10230-F	TGTCAGATTCCGCTGTCCT	Part of ZY05719_RS10230
ZY05719_RS10230-R	GCTTTTCAATAGCAACTCCT	Part of ZY05719_RS10230
lysM′-F	ATCTGCTACACGCTCTTGG	Part of *lysM′*
lysM′-R	CGTAACGGTATCTACATTGGT	Part of *lysM′*
ZY05719_RS10240-F	CCTTCTGAATAAGATTTCCCTC	Part of ZY05719_RS10240
ZY05719_RS10240-R	CTTGCGTTTTGTACCGACC	Part of ZY05719_RS10240
16S RNA-F	GTTGCGAACGGGTGAGTAA	Part of *16S RNA*
16S RNA-R	TCTCAGGTCGGCTATGTATCG	Part of *16S RNA*

a *Underlined portions of the primers correspond to restriction enzyme recognition sites. GTCGAC, Sal I; GGATCC, BamH I; and GAATTC, EcoR I*.

The upstream and downstream flanking sequences of the DNA fragment to be deleted were amplified utilizing two pairs of primers, UF/UR and DF/DR, by PCR. Then, they were integrated together by PCR using primer pair UF/DR. After digestion with restriction endonucleases (TaKaRa, *Eco*R I, Cat.No.1040S; *Bam*H I, Cat.No.1010S; *Sal* I, Cat.No.1080S), the PCR products were ligated into pSET4s (Takamatsu et al., 2001a) with T4 DNA Ligase (TaKaRa, Cat.No.2011A). Recombinant vectors were electroporated into wild-type ZY05719. Transformants were cultured at 37°C on TH agar in the presence of spectinomycin. A single colony was subjected to serial passages at 28°C in TH broth free from spectinomycin. Subsequently, the bacterium solution was diluted and plated on TH agar. Spectinomycin-sensitive colonies were verified for deletion of a particular DNA fragment by PCR using primer pairs UF/DR and F/R as well as product sequencing.

The DNA fragment containing a whole ORF of a deleted gene and its promoter sequence was amplified by PCR using primer pair CF/CR and cloned into pSET2 (Takamatsu et al., 2001b). Verified recombinant vector was electroporated into the mutant to construct the complementation strain.

### RNA manipulation and real-time quantitative PCR (qPCR)

The wild-type SS2 strain ZY05719, its derived mutants, and complementation strains were cultured to the mid-log phase (at an OD_600_ of 0.6). Total RNA was extracted using RNAiso Plus (TaKaRa, Cat.No.D9108A) following its manufacturer's instructions. The remaining genome was eliminated using DNase I (Fermentas, Cat.No.EN0521). cDNA synthesis was completed using the PrimeScript™ RT reagent kit (TaKaRa, Cat.No.RR037A) and mRNA levels were measured with the SYBR® Premix Ex Taq™ kit (TaKaRa, Cat.No.RR420A) according to the manufacturer's protocol. Primers used for the various qPCR assays are listed in Table 2. The *16s RNA* gene was used as the reference gene (Wang et al., 2011). Relative changes in gene transcriptional levels were calculated utilizing the comparative CT method (Livak and Schmittgen, 2001). Each set of qPCR assay was repeated three times with independent RNA preparations.

### Phagocytosis and intracellular survival assays of SS2 against microglial cells

Phagocytosis assays were performed as described before with some modifications (Meijerink et al., 2012; Redlich et al., 2012). BV2 cells grown to approximately 5.0 × 10^5^ cells/well in a 24-well plate were used for assays. Bacteria grown to the log-phase were harvested by centrifugation (5,000 g, 5 min), washed with and resuspended in DMEM, and added to cells at a multiplicity of infection (MOI) of 1. After 1 h of incubation at 37°C under 5% CO_2_, cells were washed three times with phosphate buffered saline (PBS) and were added to DMEM containing 5 μg mL^−1^ penicillin and 100 μg mL^−1^ gentamicin for another 1 h of incubation to kill extracellular bacteria. Then, after another three washes with PBS, cells were lysed with double-distilled water to recover intracellular bacteria. Recovered bacteria were calculated using the plate count method. The phagocytic rate was calculated as the percentage of the CFU number of intercellular bacteria recovered in the CFU number of initial inoculum. Assays were repeated as three independent experiments.

Intracellular survival analysis in microglial cells was performed based on previous reports (Cybulski et al., 2008; Tang et al., 2012) with some modifications. Briefly, the protocols were the same as the phagocytosis assay except that the incubation time after the cells were added to DMEM containing antibiotics were 1 and 3 h, respectively. Intercellular bacteria were recovered after each time point of incubation and calculated. The survival rate was calculated as CFU_3h_/CFU_1h_ × 100%.

### Transmission electron microscopy

For visual observation of intracellular SS2, samples were fixed with 2.5% glutaraldehyde and 2% formaldehyde in cacodylate buffer (0.01 M MgCl_2_, 0.01 M CaCl_2_, 0.09 M sucrose, 0.1 M cacodylate, pH 6.9) for 3 h at 4°C, washed with cacodylate buffer and treated with 1% osmium tetroxide in cacodylate buffer for 1 h at room temperature (Hammerschmidt et al., 2005). Then samples were dehydrated with a graded series of ethanol, embedded in an epoxy resin, sectioned, and counterstained with uranyl acetate. After air-drying, samples were observed on a Hitachi H7650 transmission electron microscope (Hitachi, Tokyo, Japan) at a magnification of 1,200 × and an acceleration voltage of 80 kV.

### Survival assays of SS2 in whole blood, H_2_O_2_, and acidic conditions

Survival assays in whole blood were performed as described earlier (Bonifait et al., 2010; Zhu et al., 2011). Blood samples were collected from healthy pigs and used immediately. Bacteria were cultured to an OD_600_ of 0.6, collected by centrifugation (5000 g, 5 min), and washed and resuspended in PBS to an OD_600_ of 0.2. Then, 1 mL of whole blood and 100 μL of bacteria in PBS were mixed together and incubated for 3 h at 37°C with occasional gentle shaking. After 0 or 3 h of infection with SS2, whole blood samples were diluted and plated on TH agar to determine the bacterial survival. The time point of 0 h was considered as 100 percent viability. Tests were performed three times.

The survival abilities of SS2 in hydrogen peroxide and acidic conditions were determined as described elsewhere with some modifications (Wen et al., 2011; Tang et al., 2012; Fulde et al., 2014). Briefly, bacteria grown to an OD_600_ of 0.6 were harvested by centrifugation and washed three times with 0.1 M PBS (pH 7.4). Then, the pellets were resuspended in 0.1 M PBS at a pH of 5 for 1 h of incubation at 37°C for survival assays in the acidic condition, or in 0.1 M PBS (pH 7.4) containing 5 mmol/L H_2_O_2_ for 2 h of incubation at 37°C for survival assays in the peroxidation environment, respectively. The pellets were resuspended in 0.1 M PBS at a pH of 7.4 for 1 h or 2 h of incubation at 37°C was used as negative controls. Results were recorded as the percent survival compared with the initial inoculum. Each assay was performed three times.

### Measurement of nitric oxide production

BV2 cells were stimulated with killed bacteria as previously reported with some modifications (Segura et al., 2004; Houde et al., 2012). After overnight growth, bacteria were washed with PBS and heat killed at 60°C for 45 min. Killed bacteria from 1 × 10^9^ CFU were incubated with BV2 cells (5 × 10^5^ cells/well) that had previously been plated in 24-well plates. The culture supernatants were collected after 6, 12, 24, and 48 h, respectively. Production of nitric oxide (NO) by BV2 cells was measured with a Nitric Oxide Assay Kit (Beyotime, Cat.No.S0021) according to the manufacturer's instructions. Briefly, 50 μL of Griess Reagents I and II was added in turns to the collected 50-μL supernatant samples. Absorbance at OD_540_ was spectrophotometrically determined. The NO concentration was calculated using the standard curve method with different concentrations of NO diluted in DMEM containing 10% FBS. Assays were repeated three times.

### Cytokine detection by ELISA

Cytokines were detected using an enzyme-linked immunosorbent assay (Kim and Weiser, 1998) as reported earlier with some modifications (Segura et al., 1999; Dominguez-Punaro et al., 2010). The SS2 wild-type strain ZY05719, mutant and complementation strains were added to BV2 at an MOI of 2 for 12 h at 37°C under 5% CO_2_. Commercial ELISA kits (Boster, TNF-α (m) ELISA kit, Cat.No.EK0525, IFN-γ (m) ELISA kit, Cat.No.EK0375) were used to measure the production of TNF-α and IFN-γ in the harvested culture supernatant.

### Statistical evaluations

Each experiment was repeated three times, and every treatment group in each time of test was set up three biological repetitions. The data were collected for analyzing and plotting using GraphPad Prism (version 5.0; GraphPad Software, [http://www.graphpad.com]) as reported earlier (Weissgerber et al., 2015). Data are presented as means with standard deviations (SDs). Data statistical analyses for all pairwise comparisons were assessed using Student's *t*-test. Differences were considered to be significant for a *P* < 0.05.

## Results

### Construction of deletion mutations and complementation strains

The insertion mutant of SS2 ZY05719 *hsdS* gene mentioned in the introduction was named M*hsdS*. A complete genome sequencing of M*hsdS* was performed, and there is no DNA sequence change when compared with wild-type ZY05719 except the insertion mutation of *hsdS* gene (data not shown). To get a better insight of a series of trait changes, which were as results of the mutation of *hsdS* gene, the complementation strain that M*hsdS* containing pSET2::*hsdS* was successfully constructed and named CM*hsdS*. As the *hsdS* belongs to the Type I R–M system SsuZY05719II, we constructed a *hsdS*′ deletion mutant and its complementation strain, which were named Δ*hsdS*′ and CΔ*hsdS*′, respectively, to analyze the difference in the functional impact of these two genes.

**Figure 2:**
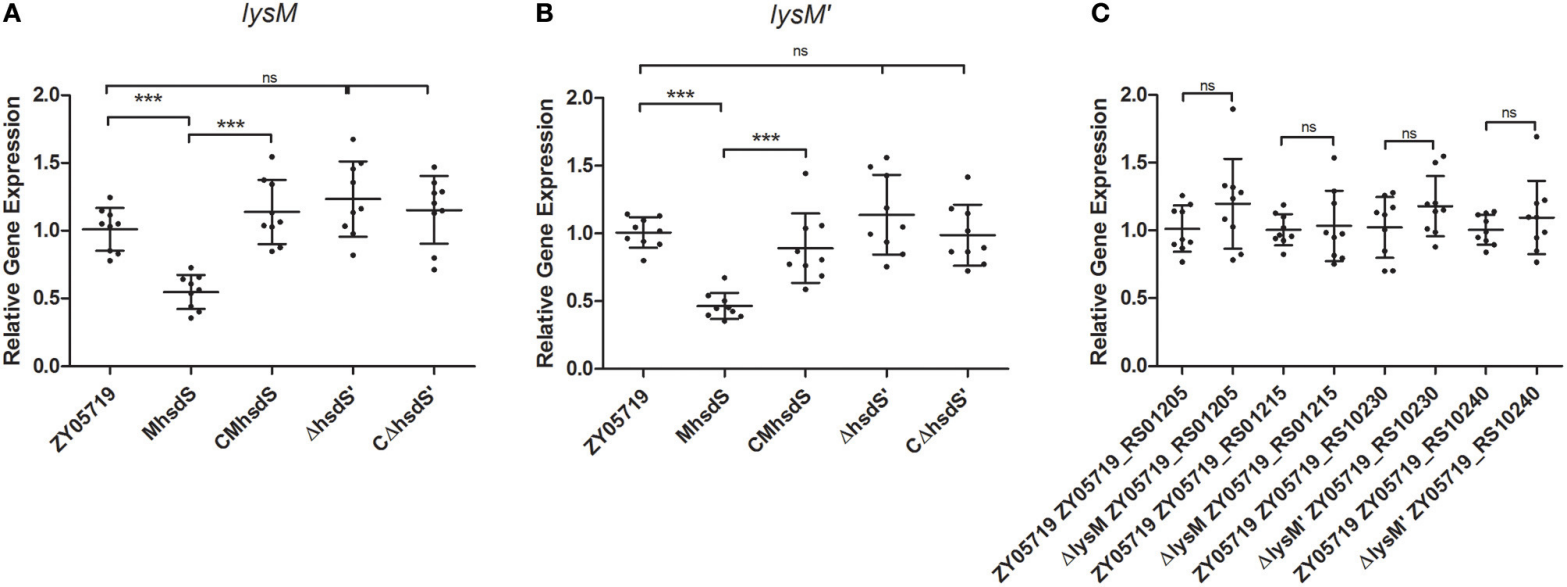
qPCR assays. **(A,B)** Relative mRNA levels of *lysM* or *lysM*′ in wild-type ZY05719, its mutants and complementation strains grown to the mid-log phase. **(C)** Relative transcriptional levels of upstream and downstream genes of *lysM* or *lysM*′ of wild-type ZY05719 and Δ*lysM*, or and Δ*lysM*′. Student's *t*-test was performed (^***^p < 0.001; ns, no significance).

Meanwhile, RNA samples of wild-type ZY05719 and M*hsdS* grown to the mid-log phase were extracted and sent to Sinogenomax in China for RNA sequence and gene transcription analysis (Table S1). As a result, the transcriptional levels of both gene peptidoglycan-binding proteins *lysM* (locus_tag ZY05719_RS01210) and *lysM*′ (locus_tag ZY05719_RS10235) were more than two-fold decreased when M*hsdS* was compared with wild-type ZY05719. Then, the changes in the transcriptional levels of these two genes were shown to be significant, and the transcriptional regulation of these two genes was impacted by *hsdS*, not by *hsdS*′, according to qPCR assays (*p* < 0.001, Figures 2A,B). We constructed *lysM, lysM*′ and double gene deletion mutants, which were named Δ*lysM*, Δ*lysM*′ and Δ*lysM*Δ*lysM*′, respectively. As both *lysM* and *lysM*′ were located downstream of the ORF of their upstream and downstream genes from the transcription direction (Figures 1B,C), the gene deletions did not theoretically affect the transcription of upstream and downstream genes; these observations were confirmed using qPCR assays, which showed there were no significant differences between the wild-type SS2 and the *lysM* or *lysM*′ deletion mutants on the transcript levels of their corresponding upstream and downstream genes (Figure 2C). As a result, the complementation strains of Δ*lysM* and Δ*lysM*′ did not need to be constructed.

There was no difference in the growth kinetics among wild-type ZY05719, its insertion or deletion mutations, and the complementation strains mentioned above (Figure S1).

### hsdS, lysM, and lysM′ contribute to the anti-phagocytosis and survival in BV2 cells and whole blood

**Figure 3:**
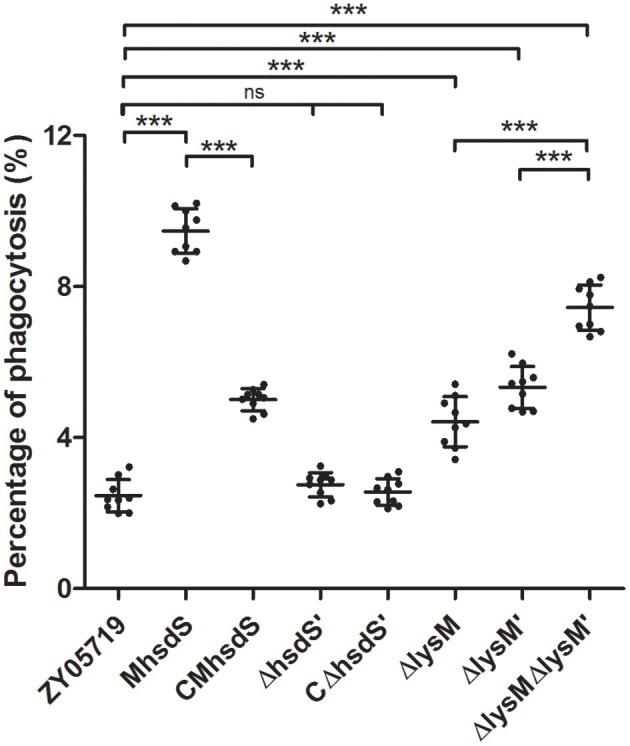
Anti-phagocytosis assays of SS2. The scatterplot showed the percentage of the number of CFUs of recovered SS2 from phagocytosis by BV2 out of the total CFUs of bacteria incubated with BV2. The statistical significance is assessed using a Student's *t*-test (^***^P < 0.001; ns, no significance).

**Figure 4:**
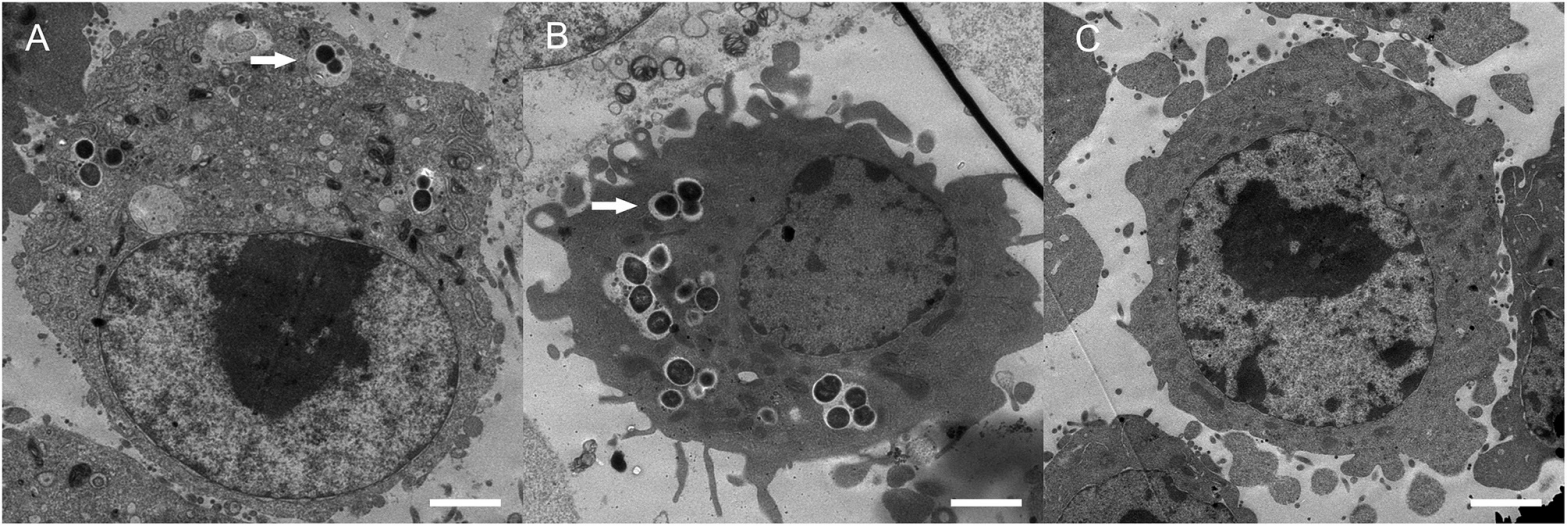
Transmission electron microscopic analyses. BV2 cells infected with wild-type ZY05719 **(A)**, M*hsdS* **(B)**, and uninfected BV2 cells **(C)** were prepared and viewed using a transmission electron microscope. Arrows indicated engulfed bacteria. Bars = 2 μm.

Phagocytosis assays showed the phagocytic rates of BV2 cells to wild-type SS2, M*hsdS*, and CM*hsdS* were 2.46 ± 0.42%, 9.47 ± 0.58%, and 5.00 ± 0.29%, respectively (Figure 3). Compared with M*hsdS*, the phagocytic rates to wild-type SS2 and CM*hsdS* were reduced 74.0 and 47.2% (*p* < 0.001), which demonstrated *hsdS* was beneficial to SS2 against phagocytosis. The result of transmission electron microscopy also revealed that the mutation of *hsdS* made the bacteria more easily to be phagocytized by a phagocyte (Figure 4). Then, we analyzed the relationship between the function of *hsdS*′ and the anti-phagocytosis of SS2. As a result, there was no significant difference between wild-type ZY05719 and Δ*hsdS*′. Meanwhile, in comparison with the wild-type SS2, the phagocytic rates to Δ*lysM*, Δ*lysM*′ and Δ*lysM*Δ*lysM*′ were significantly increased (*p* < 0.001). In addition, the phagocytic rate to Δ*lysM*Δ*lysM*′ was significantly increased (*p* < 0.001) when compared with Δ*lysM* or Δ*lysM*′.

**Figure 5:**
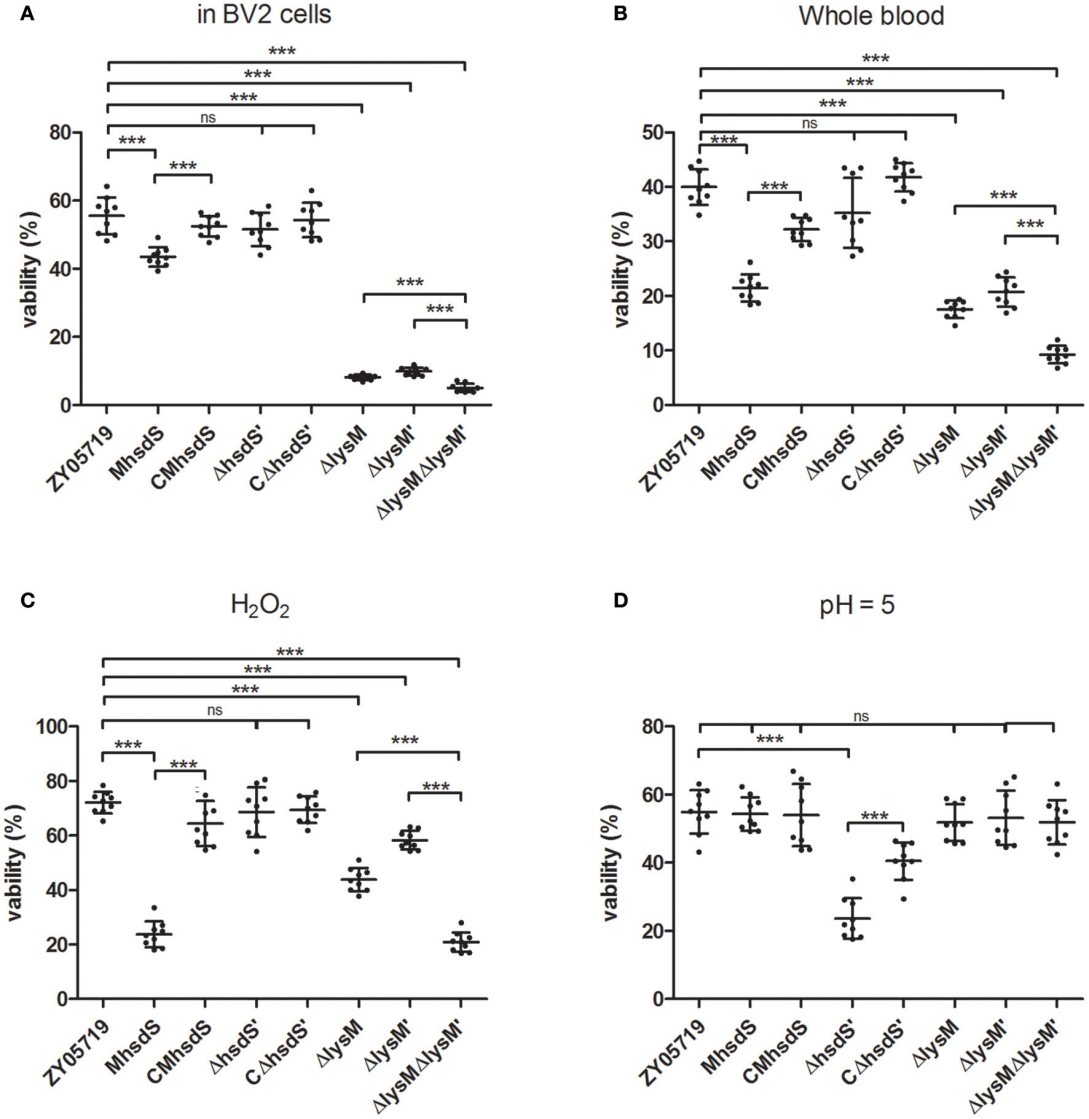
Survival assays. Wild-type ZY05719, M*hsdS*, CM*hsdS*, Δ*hsdS*′, CΔ*hsdS*′, Δ*lysM*, Δ*lysM*′, and Δ*lysM*Δ*lysM*′ grown to the mid-log phage were harvested and used to test the survival ability in BV2 **(A)**, whole blood **(B)**, a peroxidation environment **(C)** and acidic conditions **(D)**, respectively. Data represent means and standard deviations of three independent experiments. Statistical significance is indicated for Student's *t*-test (^***^p < 0.001; ns, no significance).

Next, we analyzed the influence of insertion mutation of *hsdS* on survival in phagocytes. The results showed that the survival rate of M*hsdS* in BV2 cells was significantly reduced when compared with wild-type ZY05719 or CM*hsdS* (*p* < 0.001, Figure 5A). The deletion of *hsdS*′ did not affect the survival of ZY05719 in BV2. Interestingly, the mean survival rates of Δ*lysM*, Δ*lysM*′ and Δ*lysM*Δ*lysM*′ were less than 10%, and they were significantly reduced by 85.4, 82.2, and 90.9% (*p* < 0.001) when compared with wild-type ZY05719, respectively. Still, the survival ability of double gene deletion mutant Δ*lysM*Δ*lysM*′ was significantly decreased (*p* < 0.001) when compared with Δ*lysM* or Δ*lysM*′.

The survival ability of wild-type ZY05719, its mutants and complementation strains in whole swine blood was determined (Figure 5B). The deletion of *hsdS*′ did not affect the survival ability of ZY05719 in whole blood. The insertion mutation of *hsdS*, as well as the deletion of *lysM* or/and *lysM*′, significantly reduced the survival rates of bacteria in whole blood compared with parent wild-type ZY05719 (*p* < 0.001). In comparison with M*hsdS*, the survival rate of the complementation strain, CM*hsdS*, was significantly increased (*p* < 0.001).

### Survival assays in the peroxidation environment and the acidic condition

To gain a better insight into mechanisms against the host environment that were influenced by *hsdS, lysM* and *lysM*′, other survival assays in the special environment *in vitro* were evaluated. The survival rates of all mutants and complementation strains in 0.1 M PBS at a pH of 7.4 when compared with wild-type ZY05719 were not significantly different (Figure S2). The results of survival assays in PBS containing H_2_O_2_ showed the survival rates of M*hsdS*, Δ*lysM*, Δ*lysM*′ and Δ*lysM*Δ*lysM*′ were significantly decreased when compared with wild-type ZY05719 (Figure 5C, *p* < 0.001). Meanwhile, the Δ*lysM*Δ*lysM*′ significantly decreased the survival rate when compared with Δ*lysM*, or Δ*lysM*′ (*p* < 0.001). There were no significant differences in the survival rates among wild-type ZY05719, CM*hsdS*, Δ*hsdS*′ and CΔ*hsdS*′ in PBS with H_2_O_2_, which indicated that the deletion of *hsdS*′ did not impact the survival ability of ZY05719 in the peroxidation environment.

Among all mutants, only the Δ*hsdS*′ significantly decreased the survival rate in acidic conditions (Figure 5D, *p* < 0.001) when compared with wild-type ZY05719. Additionally, the CΔ*hsdS*′ significantly recovered this rate when compared with Δ*hsdS*′ (*p* < 0.001). This result revealed that bacterial resistance to the acid environment is regulated by *hsdS*′, not by *hsdS, lysM*, or *lysM*′, which was different from the aforementioned survival assays.

### hsdS contributes to inhibition of SS2-induced NO and TNF-α production in mouse microglia

**Figure 6:**
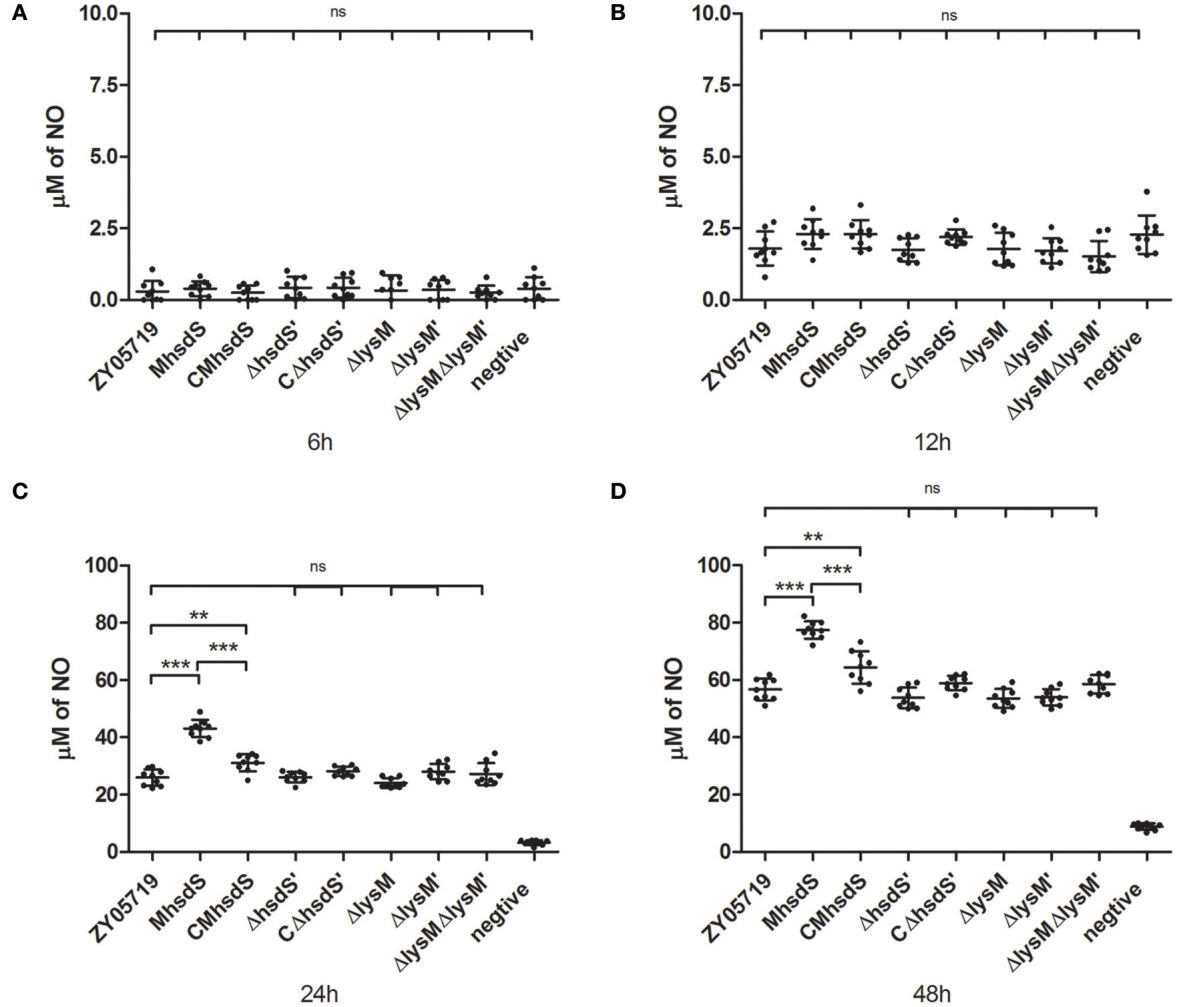
Time course of increased nitric oxide production. Wild-type ZY05719, its mutants, and complementation strains were incubated with BV2, respectively. The nitric oxide in the culture, which was produced by BV2, was measured after 6 **(A)**, 12 **(B)**, 24 **(C)**, and 48 **(D)** h of incubation. The results are given as means and standard deviations of three independent experiments. Statistical significance of pairwise comparisons was determined using Student's *t*-test (^**^p < 0.01; ^***^p < 0.001; ns, no significance).

Nitric oxide, which is another antibacterial substance produced by phagocytes after incubation with SS2, was measured as time progressed. As a result, significantly increased NO production by bacteria stimulation appeared in the 12- to 24-h time period of incubation (Figure 6). Of all mutants and complementation strains, only the M*hsdS* and the CM*hsdS* significantly increased the NO production relative to wild-type ZY05719 after 24 and 48 h of incubation (*p* < 0.01, Figures 6C,D). The NO production, which was stimulated by CM*hsdS*, was significantly less than M*hsdS* after 24 or 48 h of incubation (*p* < 0.001, Figures 6C,D). These results indicated *hsdS*′, *lysM*, and *lysM*′ were not involved in the inhibition of NO production by phagocytes.

**Figure 7:**
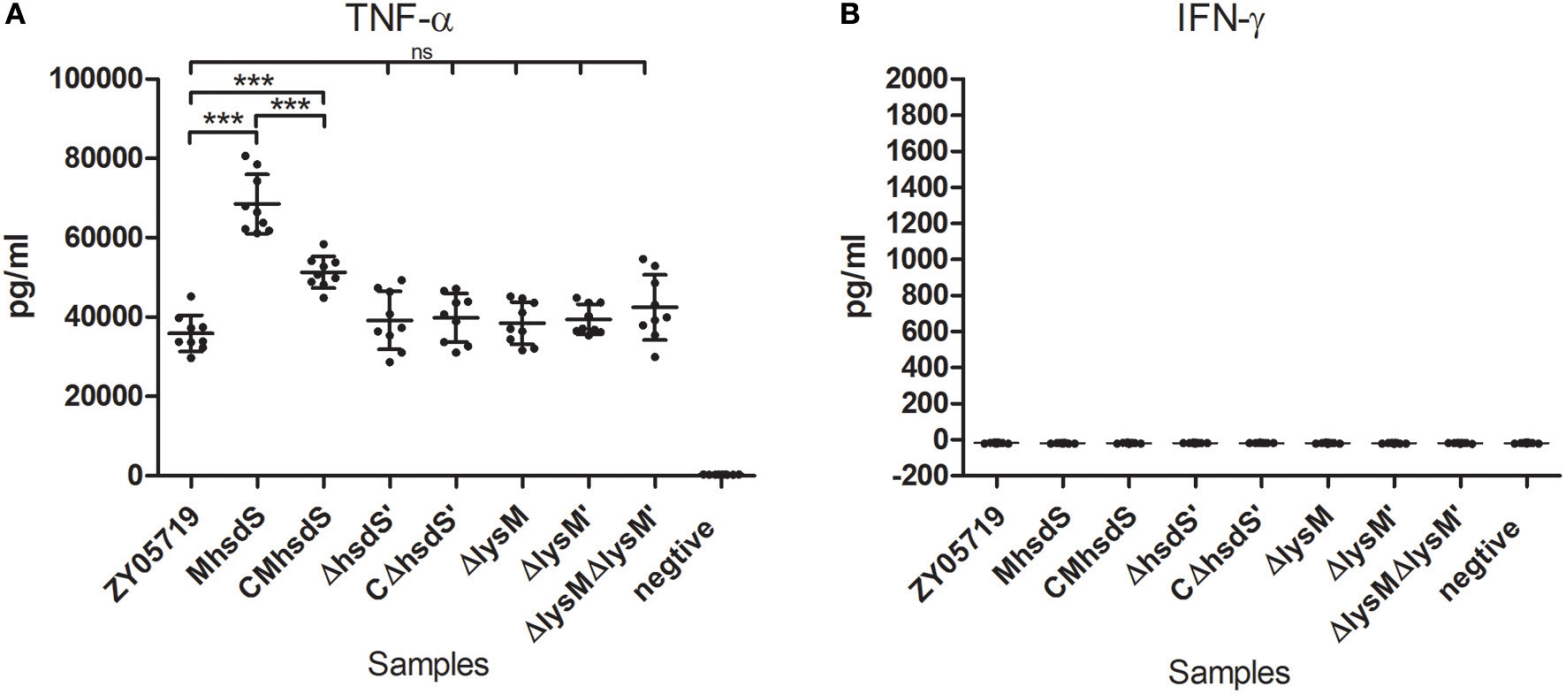
Comparative study of cytokine production. BV2 cells were incubated with different SS2 strains at a MOI of 2. The culture supernatants were harvested after 12 h of incubation and analyzed for TNF-α **(A)** and IFN-γ **(B)** production by ELISA. The data represent means plus standard deviations from three independent experiments. Statistical significance was determined using Student's *t*-test (^***^p < 0.001; ns, no significance).

It was previously reported that tumor necrosis factor-alpha (TNF-α) and gamma-interferon (IFN-γ) act synergistically to induce the NO production by phagocytes (Ding et al., 1988). Therefore, the production of these two cytokines in BV2 cells was measured after 12 h of incubation with SS2. As a result, after they were infected with SS2, BV2 cells produced significantly higher levels of TNF-α (Figure 7A, *p* < 0.001) than un-infected cells. M*hsdS* significantly increased the induced levels of TNF-α compared to wild-type ZY05719 and CM*hsdS* (*p* < 0.001). On the other hand, the deletion of *hsdS*′, *lysM*, and *lysM*′ did not have a significant effect on the TNF-α production. BV2 cells did not produce INF-γ with or without SS2 infection (Figure 7B).

## Discussion

Engulfment and subsequent degradation of pathogens are key processes in the innate immune response, as well as promote antigen presentation for developing acquired immunity. Effective phagocytosis requires two components, internalization of pathogens and maturation of the phagosome. The phagosome is an intracellular vacuole structure wrapped in plasma membrane where pathogens are usually located (indicated by arrows in Figure 4). A fully matured phagosome, the phagolysosome, is highly acidic and rich in reactive oxygen and nitrogen species (ROS and RNS) as well as multiple antimicrobial proteins and peptides. The phagolysosome has completely bacteriostatic, bactericidal and hydrolysis activity (Basset et al., 2003; Haas, 2007; Flannagan et al., 2009). On the other hand, pathogenic bacteria interfere with the internalization of phagocytic cells, survival in these cells, even in the blood and all kinds of adverse environments in the host, inducing inflammation, and diffusion to other tissues and organs, which can contribute to disease progression (Baums and Valentin-Weigand, 2009).

The S subunit is one of three types of subunits in the Type I RM system. The remaining two subunits are the M and R subunits. Under most conditions, these subunits combined into a pentameric protein complex, 2R+2M+S. In some type I RM systems, a trimer of 2M+S solely acts as a methyltransferase (Loenen et al., 2013). However, the restriction endonuclease activity requires additional two R subunits in the pentamer (Loenen et al., 2013). The activities of the two enzymes are indispensable to a series of functions in the type I RM system. Multimers both present the S subunit, which plays a role in target recognition. In addition, we demonstrated there is no exchange of TRDs between *hsdS* and *hsdS*′ in the Type I RM system SsuZY05719II, which was mentioned in the introduction. Hence, the insertion mutation in one of the TRDs in *hsdS* leads to the complete dysfunction of *hsdS* with subsequent loss of the partial regulating function of the Type I RM system SsuZY05719II. The influenced target DNA was recognized by *hsdS*, but it was not recognized by an inserted TRD accompanied by another TRD from *hsdS*′.

In our study, we found that mutation of *hsdS* made the SS2 more easily internalized and degraded by microglia, more quickly eliminated in whole blood, more susceptible to peroxidation environment, and more inducible with secretion of NO and TNF-α by microglia. However, there is no significant difference between wild-type ZY05719 and M*hsdS* in the resistance to an acidic environment. Except for the role of *hsdS* in anti-phagocytosis, these results also suggested regulation mechanisms for facilitating survival in phagocytes and whole blood controlled by *hsdS*, were enhancing the bacterial capacity of resistance to ROS and inhibition of the production of host-derived RNS. Meanwhile, *hsdS*′ deletion made the SS2 more susceptible to acidic conditions, which revealed genes impacted by the type I RM system multimer containing *hsdS*′ are involved in resistance against the acid environment. However, the survival rates of Δ*hsdS*′ in BV2 and whole blood did not significantly reduced compared with wild-type ZY05719, which indicated only weaken acid resistance is at least not enough to reduce the survival ability of SS2 in murine microglia and swine whole blood during the test period. These findings indicate significance of the regulating function of the type I RM system in bacterial resistance on bacteriostatic and bactericidal activity in phagocytes and whole blood.

To explore the regulation mechanism of *hsdS* on SS2, the differences in the transcription level of the wild-type SEZ and M*hsdS* were analyzed by transcriptome analysis and qPCR verification. We found the loss of *hsdS* led to down-regulation of the transcript levels of two peptidoglycan-binding proteins, which both harbor one lysM domain at the N terminus. Subsequently, we observed deletion of *lysM* and *lysM*′, especially the double gene deletion of them significantly reduced the anti-phagocytic ability and survival ability in phagocytes, whole blood, and PBS with H_2_O_2_ from SS2. This demonstrated the positively influence of *hsdS* on transcription of *lysM* and *lysM*′ is at least one mechanism that helps *hsdS* facilitate SS2 with anti-phagocytosis and survival against adverse host environments. Meanwhile, *lysM* and *lysM*′ were not involved in acidic resistance, which corresponded to the observation that *hsdS*′ was not involved in the transcript regulation of these two genes. The regulatory mechanisms on the survival in acidic conditions of SS2 by *hsdS*′ require further research. The product of *lysM* only exhibits 37% amino acid identity in the lysM domain with that of *lysM*′, and the remaining amino acid sequences of these two proteins show no identity, implying the function of these two proteins may be very different.

An earlier study from our laboratory discovered a peptidoglycan-binding protein (locus_tag BFP66_RS01040) containing the lysM domain of *Streptococcus suis* serotype 9 GZ0565 (GenBank Accession number NZ_CP017142) facilitates survival of SS in whole blood through releasing more free iron from the host (Wu et al., 2016). The product of *lysM* exhibits 82% amino acid identity with peptidoglycan-binding protein BFP66_RS01040. Fe^2+^ is a cofactor of microbial housekeeping enzymes. The host used such as lactoferrin, natural resistance-associated macrophage protein 1 in the phagosome (Flannagan et al., 2009), as well as transferrin, lactoferrin, and ferritin in blood (Daou et al., 2009) to create iron-limited environments on bacteriostatic activity. The ability of bacteria to exploit free iron from their hosts, which contributes to bacterial survival, can be an explanation of pathogenesis of *hsdS*.

Other aspects, we found only M*hsdS*, and not Δ*hsdS*′, Δ*lysM*, Δ*lysM*′ or Δ*lysM*Δ*lysM*′, induced more NO and TNF-α production in BV2 cells than wild-type SS2. NO has been implicated in the cellular toxicity in many cell systems, including the brain (Madrigal et al., 2002), but it also acts a major antimicrobial mechanism (Fang, 1997). It is reported that TNF-α induces NO production via NF-κB activation (Lamas et al., 1991; Madrigal et al., 2002). The reducing of TNF-α production can be regulated by *hsdS*, one of the mechanisms for reducing NO production, which in the end enhanced the survival ability of SS2 in phagocytes and whole blood.

IFN-γ, another NO product inducer (Ding et al., 1988; Flynn and Chan, 2001), was reported to be secreted in mouse and human lung macrophages with the infection of *Mycobacterium tuberculosis* (Fenton et al., 1997; Wang et al., 1999). However, to the best of our knowledge, there is no relevant research on the production of IFN-γ in microglial cells. Additionally, other *streptococcus* species, such as *Streptococcus bovis* and *Streptococcus pneumonia*, were reported to induce IFN-γ secretion in mice *in vivo* (Paiva et al., 2012; Kim et al., 2016). In our study, we found that incubation of SS2 failed to induce IFN-γ secretion in murine microglial cells, and the specific reasons for this are under study.

In summary, our study demonstrated that *hsdS*, which belongs to the type I RM system, facilities anti-phagocytosis and survival in microglia and whole blood of SS2 through positively impacting the expression of virulence-associated factors *lysM* and *lysM*′, enhancing the survival ability against peroxidation environment, and reducing the TNF-α and NO production in microglia. As far as we know, this research is the first systematic study on the regulation of virulence in the RM system in SS2, which may provide new insights into the pathogenesis SS2 as well as the prevention and treatment of its resulting disease.

## Author contributions

HF, BX, and PZ conceived the study; BX, PZ, WL, RL, and JT performed the experiments; BX and PZ analyzed experimental results; BX, PZ, and HF wrote the manuscript.

### Conflict of interest statement

The authors declare that the research was conducted in the absence of any commercial or financial relationships that could be construed as a potential conflict of interest.
