# Supplementary figures and images for: hsdS, Belonging to the Type I Restriction-Modification System, Contributes to the Streptococcus suis Serotype 2 Survival Ability in Phagocytes
Source: Front Microbiol. 2017 Aug 9;8:1524. doi: 10.3389/fmicb.2017.01524 (PMC5552720; doi:10.3389/fmicb.2017.01524)

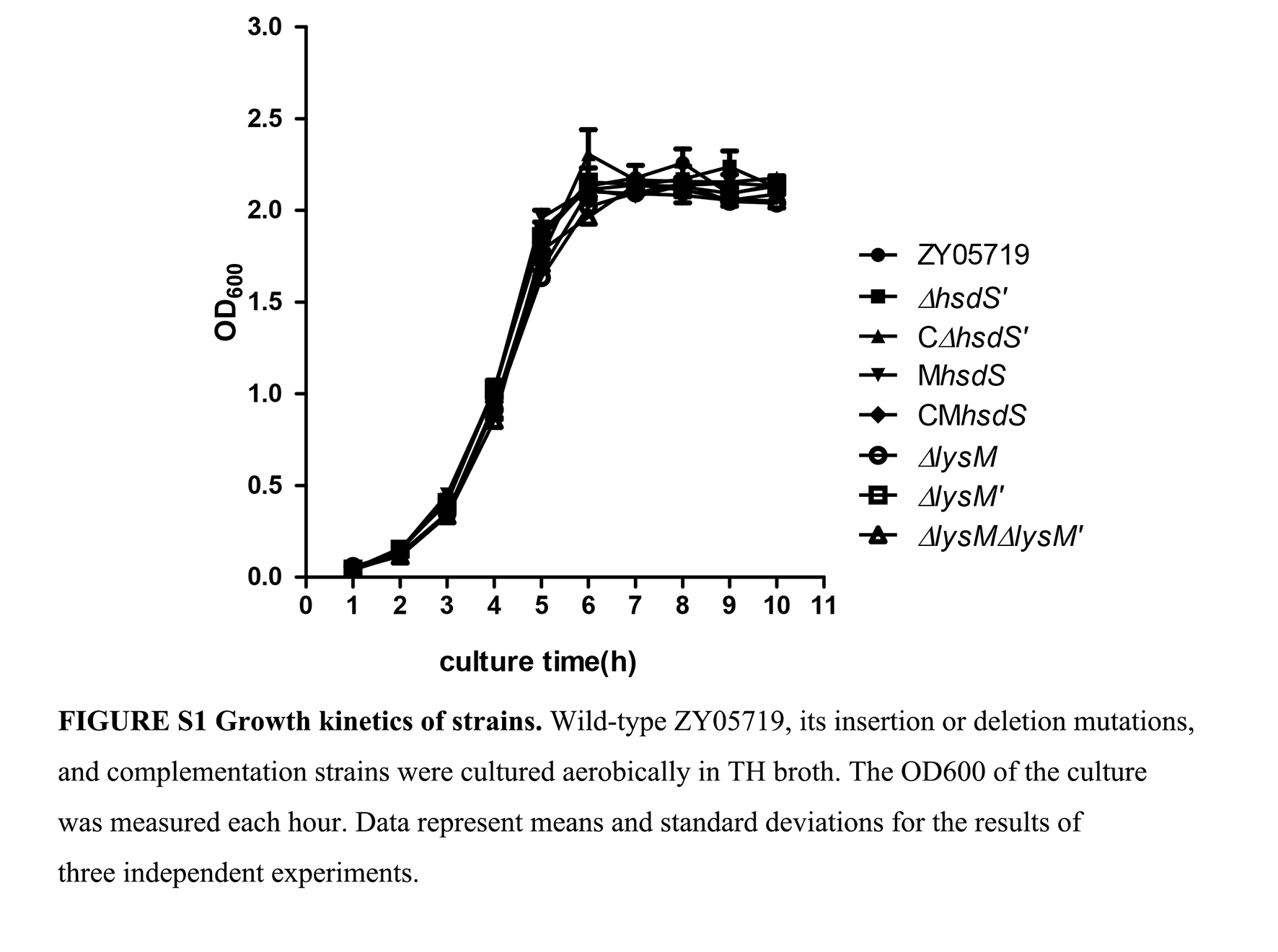

Supplement: Supplementary file 1 [file Image1.TIF]

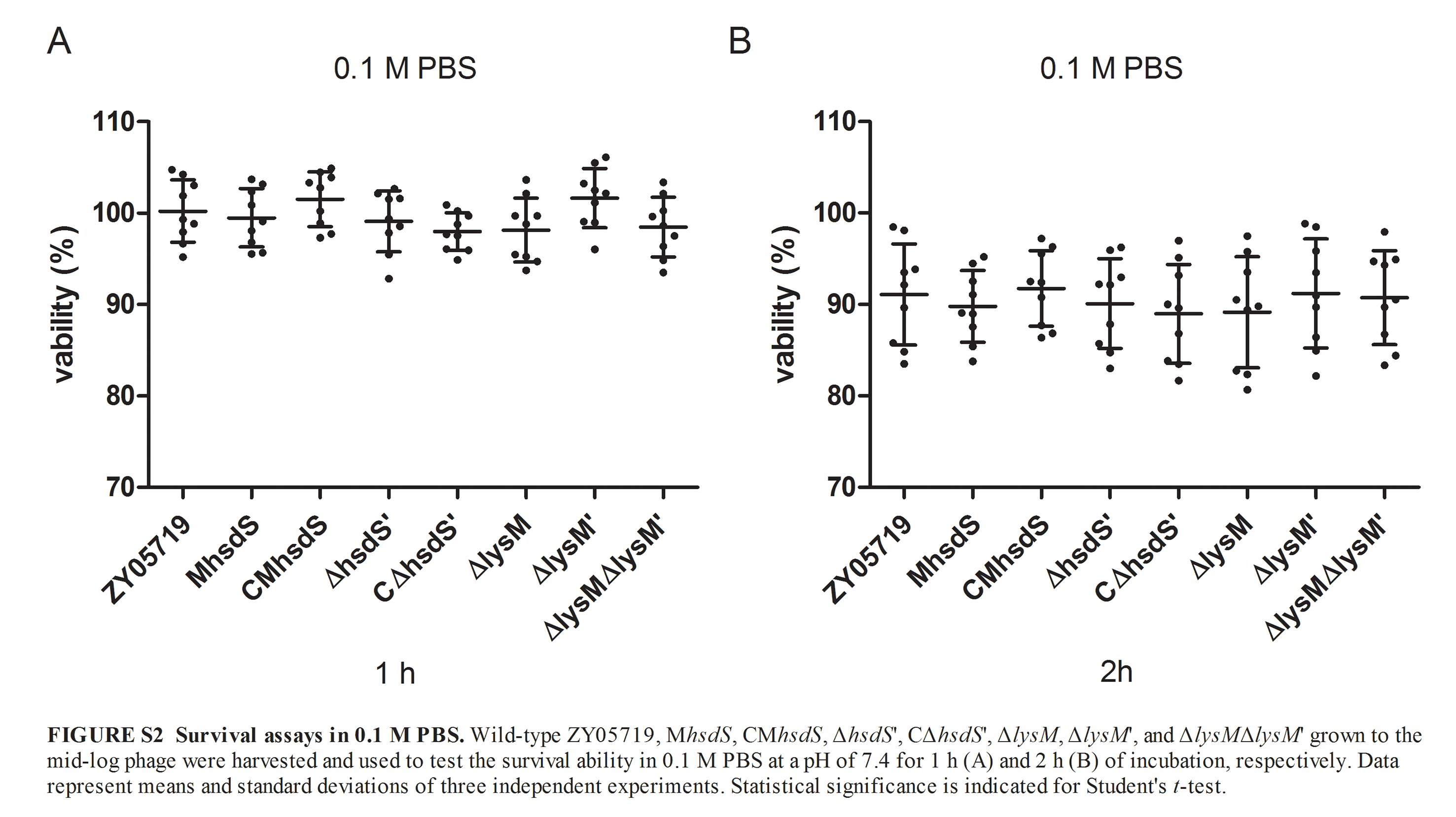

Supplement: Supplementary file 2 [file Image2.TIF]
